# Band width selection data from Near Infra-red Spectral (NIRS) quantitative modelling of energy storage components (protein, lipid, glycogen) for single and multi-bivalve species models

**DOI:** 10.1016/j.dib.2018.04.054

**Published:** 2018-04-22

**Authors:** Jill K. Bartlett, William A. Maher, Matthew B.J. Purss

**Affiliations:** a Ecochemistry Laboratory, Institute for Applied Ecology, University of Canberra, Bruce, ACT, Australia; b Pangaea Innovations Pty. Ltd., Canberra, ACT, Australia

## Abstract

Data presented in this article are related to the research article entitled “Near Infra-red spectroscopy quantitative modelling of bivalve protein, lipid and glycogen composition using single-species versus multi-species calibration and validation sets” [1]. Band width selections were determined using a data-driven approach to modelling Near Infra-red Spectra (NIRS) of protein, lipid and glycogen content in bivalves. Models were produced for single species and combined species of *Saccostrea glomerata, Ostrea angasi, Crassostrea gigas, Mytilus galloprovincialis* and *Anadara trapezia*. Band width selection was undertaken using Fourier wavelet transformation coupled with a genetic algorithm (GA) to aggregate adjacent wavelet bands to select the minimum number of IR bands that were consistently identified in the majority of individual spectra.

**Specifications Table**

**Table:** 

Subject area	Biology, chemometric quantitative modelling
More specific subject area	Bivalve energetic quantitative modelling using NIRS.
Type of data	Excel spreadsheet
How data was acquired	FT-IR spectra capture using NIRS
	Bandwidth selection using Fourier wavelet transformation coupled with a GA to aggregate adjacent wavelet bands
Data format	Raw
Experimental factors	2nd derivative and MSC correction of spectra prior to bandwidth selection
Experimental features	Data collected during data-driven quantitative modelling process. Spectral image captured for near infra-red range, pre-processed then bandwidth selection undertaken using fourier wavelet transformation and GA to aggregate adjacent wavelet bands.
Data source location	Multiple sites on Australian east coast
Data accessibility	Data is provided with this article
Related research article	Companion paper to:
	Bartlett et al. [1]

**Value of the data**

• Data provides example of different bandwidth selections associated with energy stores in bivalve species when using a data-driven approach to NIRS quantitative modelling.
• Data provides first steps to allowing potential comparisons with other NIRS bandwidth selection processes.
• Bandwidth selection was undertaken for individual species and pooled to generate 3-oyster and 5-bivalve species models.

## 1 Data

In the near infra-red range of light, absorptions correspond to overtones and combinations of fundamental bands of molecular vibrations [2]. Data analysis of NIR spectra using multi-linear regression allows for computation of predictive models [3], [4]. In undertaking regression analysis, more effective and robust correlations are obtained by applying an approach to discriminate within the spectra on which band widths to use in the quantitative modelling [4]. Band width or variable/feature selection is critical to the calibration process as it allows for improvement of data quality by including relevant information, providing better prediction results and reducing uninformative ‘noise’ Smirnov,[5].

The data provided are the results of a data-driven bandwidth selection process implemented when undertaking quantitative modelling of bivalve energy storage components of protein, lipid and glycogen in whole animals. Single species models were developed for each energy storage component for *Saccostrea glomerata*, *Ostrea angasi, Mytilus galloprovincialis* and *Anadara trapezia*. Multi-species models were developed for 3 oyster species (*S. glomerata*, *O. angasi* and *Crassostrea gigas*) and all 5-bivalve species.

## 2 Experimental design, materials, and methods

Bivalve species used in this modelling were collected across 8 sites in New South Wales (NSW), Victoria and South Australia (SA) in Australia across four seasons to provide a wide range of samples.

NIR spectra were collected with a Perkin Elmer Frontier FT-IR Spectrometer using the NIR spectral unit. Samples added to the dish were gently pressed into the dish (30 mm) then tapped three times with a spatula to ensure even packing. NIR spectra were captured at wavelengths 10,000–4000 cm^−1^ (32 scans) measured as absorbance at a resolution of 16 cm^−1^ with data intervals of 2 cm^−1^. NIR capture was undertaken in triplicate and samples rotated up to 120° between each image capture. Spectra were captured using Perkin Elmer Spectrum software, v.10.4.3.339 and corrected for stray light and reference corrected.

Data-driven software was developed to undertake all pre-processing and predictive NIR spectra model generation. Data was pre-processed by applying multiplicative scatter correction and second derivative using the mean of the triplicate NIRS scans to normalise the data. Samples were then screened for outliers with samples where the Mahalanobis distance between individual analyte concentration values and the median analyte concentration for the entire sample dataset is > 3.0 with outliers being excluded from the model datasets [3], [6]. The dataset was then segregated into calibration and validation datasets following the methods described by Jiwen et al. [4] and Zhu et al. [7]. Briefly, the data was ordered from lowest to highest with minimum and maximum values allocated to the calibration data set. The remaining samples were randomly allocated to either the calibration or validation data set with 25% allocated to the validation set and the remainders to the calibration set [4], [7]. This ensured that the calibration dataset contained the full range of the analyte concentrations and that both calibration and validation datasets contained a random selection of samples from across the entire sample dataset. Models were run up to 5 times to ensure robust and repeatable outcomes.

Band width selection was undertaken using Fourier wavelet transformation coupled with a GA to aggregate adjacent wavelet bands to select the minimum number of IR bands that were consistently identified in the majority of individual spectra. The wavenumber search range used to identify the wavelet peaks in the pre-processed spectra was between 5 cm^−1^ and 50 cm^−1^. Wavelet peaks between 2 and 100 cm^−1^ were tested, with the range of 5 to 50 cm^−1^ obtaining the most robust models.

## Appendix A Supplementary material

Lipid Band width selections_Supplementary material.

.

Protein band width selections_Supplementary material.

.

Carb Band width selections_Supplementary material.
